# Retrospective analysis of diabetic foot osteomyelitis management and outcome at a tertiary care hospital in the UK

**DOI:** 10.1371/journal.pone.0216701

**Published:** 2019-05-16

**Authors:** Mauricio Arias, Sittiga Hassan-Reshat, William Newsholme

**Affiliations:** Department of Infection, Guy’s and St Thomas’s Hospital, London, United Kingdom; Consorci Parc de Salut MAR de Barcelona, SPAIN

## Abstract

**Objectives:**

This study aimed to analyse retrospectively management and outcomes of the diabetic foot osteomyelitis (DFOM) multi-disciplinary team at St Thomas’ Hospital, London.

**Methods:**

Patients admitted during 2015 with diagnosis of DFOM were included. Data were obtained from medical and microbiology records.

**Results:**

275 patients were admitted for DF infection in 2015: 45.1% had OM (75% males). 40% were newly diagnosed with DF ulcer (DFU). 81% patients had X-ray and 28% had MRI. Bone infection was confirmed by MC&S in 53% cases. 930 microbiological isolates were obtained: 63% were Gram-positive microorganisms [*S*.*aureus* and MRSA (~40%), *CoNS* (20%), and *E*.*faecalis* (8%)]. All MRSA were vancomycin and linezolid sensitive. 23.2% isolates were vancomycin-resistant enterococci. 24% isolates were Gram-negative organisms: *P*.*aeruginosa* (42%), *E*.*coli* (13%), and *E*.*cloacae* (12%). Meropenem resistance was low; 5.4% *P*.*aeruginosa*, 87.5% *A*.*baumanii*. 76% patients received co-amoxiclav; 41% received ≥3 antibiotics; 17% received >3 months antibiotics. Hospital mean-length of stay was 26.1 days. Ulcer time-to-heal was >6 months in 25% patients. 22% ulcers healed without surgery, 60% healed after minor amputation, 12% patients had major amputation.

**Conclusion:**

Despite current MDT approach, many patients progress to amputation. DF-OM still represents a challenging clinical condition, requiring further study to develop better management guidelines.

## Introduction

The global estimate of adults living with diabetes in 2014 was 422 million, compared to 108 million in 1980. Diabetes caused 1.5 million deaths in 2012, with an estimated worldwide direct annual cost above US$827 billion [1]. In the UK, there were almost 2.9 million people diagnosed with diabetes in 2013 [2].

Poorly controlled diabetes associates with development of complications, such as chronic foot ulceration. Trauma, combined with reduced blood flow-associated poor healing and nerve damage (neuropathy), is thought to be the mechanism involved. It is estimated that 10% of people with diabetes will have a diabetic foot ulcer (DFU) at some point in their lives [2]. The national health system (NHS) in the UK spends about £650 million per year on DFU and amputations. A DFU can be defined as a localised injury to the skin and/or underlying tissue, below the ankle, in a person with diabetes [2]. Microorganisms inevitably colonise the wound, where they proliferate causing tissue damage and inflammation, eventually giving rise to clinical infection. These infections tend to be difficult to control leading to poor ulcer healing. They may spread locally and into deeper tissues, often reaching the bone [3]. Osteomyelitis (OM), is a frequent complication of a DFU. The proportion of mild-moderate foot infections with bone involvement is likely 10–20%, whereas this percentage is even greater in serious foot infections, 50–60% [4]. Contiguous spread of bacteria from soft tissue to bone means that cortical infection will precede marrow involvement. Thus, virtually all patients with suspected OM present with cortical involvement and features of chronic OM [5]. Among the important considerations for diagnosis and treatment of diabetic foot OM (DFOM) are: infection site (forefoot, midfoot, or hindfoot), degree of peripheral neuropathy, vascular supply, extent of soft-tissue and bone destruction, degree of systemic illness, and patient’s preferences [4]. Diagnosis of OM can be complex, but early diagnosis of diabetic foot infection is imperative to preventing complications such as amputation.

The National Institute for Clinical Excellence (NICE) in the UK has developed guidelines for diagnosis, prevention and management of diabetic foot problems, including OM [2]. These guidelines strongly suggest that all hospitals should have a care pathway for diabetic foot problems. This pathway includes a multidisciplinary team (MDT) made up of vascular surgeons, podiatrists, diabetologists, and infectious diseases specialists, which can evaluate the foot optimally within 24hours for the presence of any features of diabetic foot infection. Such evaluation should include decisions on specialist wound care, debridement, pressure off-loading, advice on infection and antibiotics, and whether surgical intervention is needed [2]. Guys and St Thomas’ NHS Foundation Trust Hospital, London has an established MDT, which adopted these guidelines and aims to have a holistic DF care pathway. Here, we analysed retrospectively the diagnosis and management of DFOM by this MDT during the year 2015.

## Materials and methods

### Population of study

Patients with DFU admitted to St Thomas’ Hospital (a tertiary-840 bed hospital located in London), during 2015 and who were assessed and followed up by the diabetic MDT were scrutinised for diagnosis of OM and selected for the study. Admission for reasons other than OM were excluded as were non-diabetic patients. Demographic and clinical data were obtained from electronic clinical and medication records (EPR, E-noting and Medchart). Such information included admission date, gender and age, diagnosis of OM, inflammatory markers, history of perivascular disease (PVD) and peripheral neuropathy (PN), history of revascularisation, wound location in the foot, history of surgical intervention and its indication, as well as discharge destination and mobility status post-discharge. Surgery was defined as that involving debridement, partial or minor amputation (single or multiple toe amputation, metatarsal amputation/excision, transmetatarsal amputation) and major amputation, which implied above or below knee amputation (AKA/BKA). The type of surgery described in Results was the last surgery the patient had that was the most radical, i.e if a patient had debridement, toe amputation and BKA, the type of surgery entered was BKA. Radiological information was obtained from picture archiving and communications system (PACS, Sectra AB, Linköping, Sweden). Microbiological data was retrieved from WinPath enterprise (CliniSys, Chertsey, UK). For some patients, follow up beyond 31^st^ December 2015 was necessary to gather information for length of stay, antibiotic duration, and DFU time-to-healing. The latter was defined by written documentation from podiatry clinic attendance. Ethical approval for the study was not required as data were anonymised and results were presented collectively.

### Diagnosis of osteomyelitis

This was taken directly from clinical notes as per MDT judgement. Diagnosis was based on a combination of clinical, radiological and microbiological criteria, the latter relying on bone biopsy rather than a simple swab. Patients were diagnosed based either on one, two or all three criteria. Some patients were diagnosed based on clinical grounds only, with the probe-to-bone test used to assist the diagnosis when applicable, although this procedure was not always documented in the patients’ clinical notes. Clinical diagnosis included, for acute OM, the presence of new sausage toe with local signs of inflammation or systemic upset. In these cases, X-ray is usually not helpful, and if there is no ulcer, probe-to-bone test is not useful. Chronic OM usually presented with a non-healing deep ulcer probing to bone, with visible cartilage or bone, or evidence of bone fragments. Biopsies were taken either on the ward by the podiatrist or in theatre by the surgeons. The latter is usually more reliable since they are taken under sterile conditions. Imaging included either X-ray or MRI or both.

White cell count (WCC) and C-reactive protein (CRP), are important markers of inflammation that support the diagnosis of infection. The values used were those obtained on admission to hospital. Results were compared between patients who had surgery and those who did not. Further analysis was performed by splitting the group of patients who had surgery into those with sepsis, and those with ischaemia. Patients with sepsis were those who had extensive tissue necrosis of the foot, suggesting wet gangrene, and had a high NEWS (national early warning score)[6, 7]. This score includes six physiological parameters, which are routinely recorded: respiration rate, oxygen saturation, systolic blood pressure, pulse rate, level of consciousness, and temperature. Those patients with a score of 5 or more were at risk of potential serious acute clinical deterioration needing an urgent clinical or surgical response. Patients with a diagnosis of ischaemia had a low NEWS and reduced local inflammatory signs, but still had evidence of soft tissue infection and/or necrosis for inflammatory markers (WCC and CRP).

Patients who had a non-healing ulcer but for whom the clinical notes were not clear as to whether this was due to sepsis or ischaemia, were excluded from the analysis.

### Data analysis

Statistics analysis was preformed using GraphPad Prism, version 4, 2003. Results are presented as the mean ± (SD). Unpaired t-test was used to compare the means of CRP and WCC between patients who had surgery and those who did not. One-way ANOVA was used to compare means between more than 2 groups with parametric distribution, with Tukey’s Multiple Comparisons test used to determine mean differences between groups. Group comparisons with non-parametric distribution were analysed by Kruskal-Wallis test, with the Dunn’s Multiple Comparisons test used to compare differences in the sum of ranks between two columns. *p*<0.05 was considered statistically significant.

## Results

### Demographics

During 2015, 275 patients were admitted to St Thomas’ Hospital with DF infection. Diagnosis of OM was made on 116 (42%) of these patients, although 8 had OM in two different foot locations totalling 124 OM cases. Twenty-eight (24%) OM patients had a repeat admission with the same ulcer, whereas 8 (7%) were re-admitted for OM at different locations. The latter were considered as new cases. OM most frequently occurred on the forefoot (114, 92%), followed by the hindfoot (10,8%) and midfoot (1%). Fifty-five patients (47%) had pre-existing ulcers, whereas 46 (40%) were newly diagnosed. Thirty-one patients (25%) were referred from another hospital. Mean age was 67.1 years, range 40–100; 87 (75%) patients were male.

### Diagnosis of osteomyelitis

Different modalities were used for the diagnosis of OM. These included the probe-to-bone (PTB) test, imaging (X-ray and MRI), biopsy, and diagnosis based on clinical grounds only.

The PTB test was documented in 31 patients (25%) with diagnosis of OM. Of these, one was confirmed by MRI, 6 by X-ray, 2 by MRI and X-ray, 11 by biopsy, and 10 by X-ray and biopsy. The commonest imaging modality used was plain X-ray, which was performed in 100 cases (81%), of which 54 (54%) had radiological evidence of OM. MRI was performed in 35 cases (28%), 23 of which showed evidence of OM either on its own (13) or in combination with X-ray (9), PTB test (1), biopsy (2), or X-ray and biopsy (2). Serial imaging was done in 34 patients (27%). Diagnosis based on clinical grounds only, as described in the Methods section, was obtained in 11 (9%) patients.

Eighteen patients whose ulcer healed without surgery were diagnosed using different modalities. Five were diagnosed based on clinical grounds only. Six were diagnosed by MRI, of which 3 also had an X-ray and 1 had the combination of MRI, X-ray and biopsy. Two patients were diagnosed by X-ray only, three had a combination of X-ray and biopsy, and one had X-ray and PTB.

### Inflammatory markers

**Fig 1:**
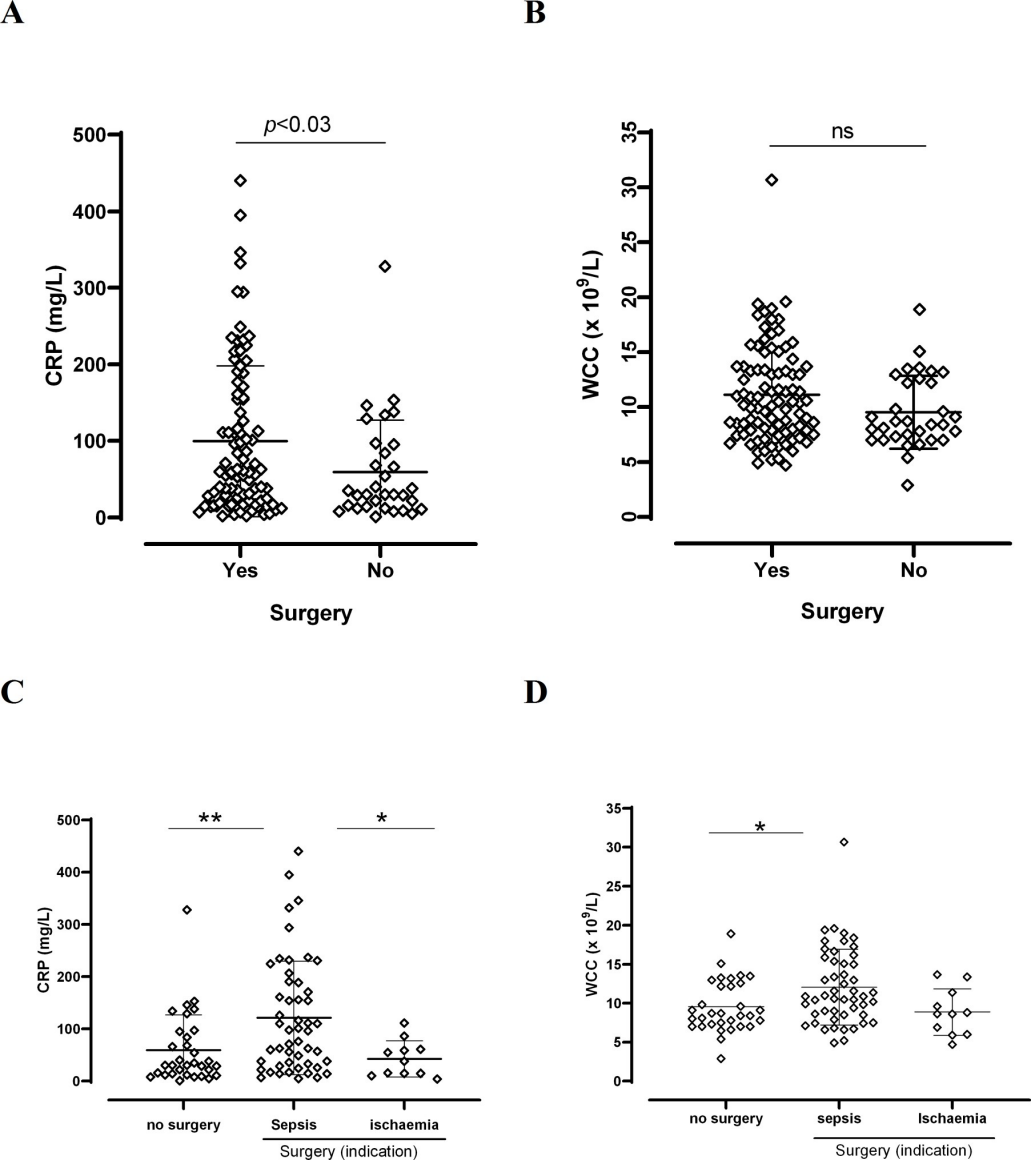
Inflammatory markers in surgical and non-surgical diabetic foot osteomyelitis. **A** and **B**: CRP and WCC, respectively, are plotted for patients who had and who did not have surgery. *n*:“Yes” 91; “No” 32. Unpaired *t*-test, significant *p*<0.05. **C** and **D**: CRP and WCC in patients who had no surgery and those who had surgery indicated by sepsis or ischaemia. *n*: no surgery: 32; surgery sepsis: 50; surgery ischaemia: 11. One-way ANOVA C: p<0.002; D: p<0.01. Tukey’s Multiple Comparison test: C: **: p<0.009; *: p<0.03; D: *: p<0.02.

Inflammatory markers on the date of admission, represented by WCC and CRP, were analysed. Comparisons were made between values for those patients who did not have surgery and those who did (Fig 1). Mean CRP was 99.7 mg/L (*n* = 91) for patients with surgery and 59.2 mg/L (*n* = 32) for patients with no surgery (*p*<0.03; 95% CI -77-7 to -3.30). Mean WCC was 11.1x10^9^/L for patients with surgery and 9.5 x 10^9^/L for patients with no surgery (*p* = ns: 95% CI -3.28 to 0.09) (Fig 1A and 1B). Role of CRP and WCC to identify those patients with sepsis, was more notable when patients who had surgery were divided according to the indication for surgery into sepsis and ischaemia. Some of the values were excluded since it was not clear from the data whether the cause of necrosis or non-healing of the ulcer was due to sepsis or ischaemia. As shown in Fig 1C and 1D, CRP and WCC were significantly higher in the sepsis group of those patients who had surgery, when compared with those who did not have surgery. CRP but not WCC was also significantly higher in the sepsis group when compared to the ischaemia group (Fig 1C and 1D). There was no association between levels of inflammatory markers and imaging (data no shown).

### Microbiological diagnosis

There were 414 microbiology samples (swab, tissue, and bone). Bone and tissue accounted for 222 samples (54%, Table 1). Sampling was analysed per patient and was represented either by a swab, tissue or bone sample/patient, or a combination of these (swab+tissue, swab+bone, tissue+bone, swab+tissue+bone) (Table 2). There were 65 (30%) swab only samples/patient out of a total of 216 samples/patient, whereas 31 (14%) and 33 (15%) were tissue only and bone only sample/patient, respectively. However, when bone sampling was taken in combination with other type of samples such as swab + bone, tissue + bone, and swab + tissue + bone, bone sampling/patient reached nearly 40% (Table 2).

**Table 1:** Type of microbiology sample for diagnosis of diabetic foot osteomyelitis.

Type of sample					
	Bone	Tissue	Swab	No sample	Total
Number	**104**	**118**	**179**	**13**	**414**
%	**25.1**	**28.5**	**43.2**	**3.1**	**100**

**Table 2:** Type of microbiology sample per patient for diagnosis of diabetic foot osteomyelitis.

Type of sample per patient								
	Swab only	Tissue only	Bone only	Swab + tissue	Swab + bone	Tissue + bone	Swab + tissue + bone	No sample
Number	**65**	**31**	**33**	**22**	**15**	**19**	**18**	**13**
%	**30.1**	**14.3**	**15.3**	**10.2**	**6.9**	**8.8**	**8.3**	**6.0**

**Fig 2:**
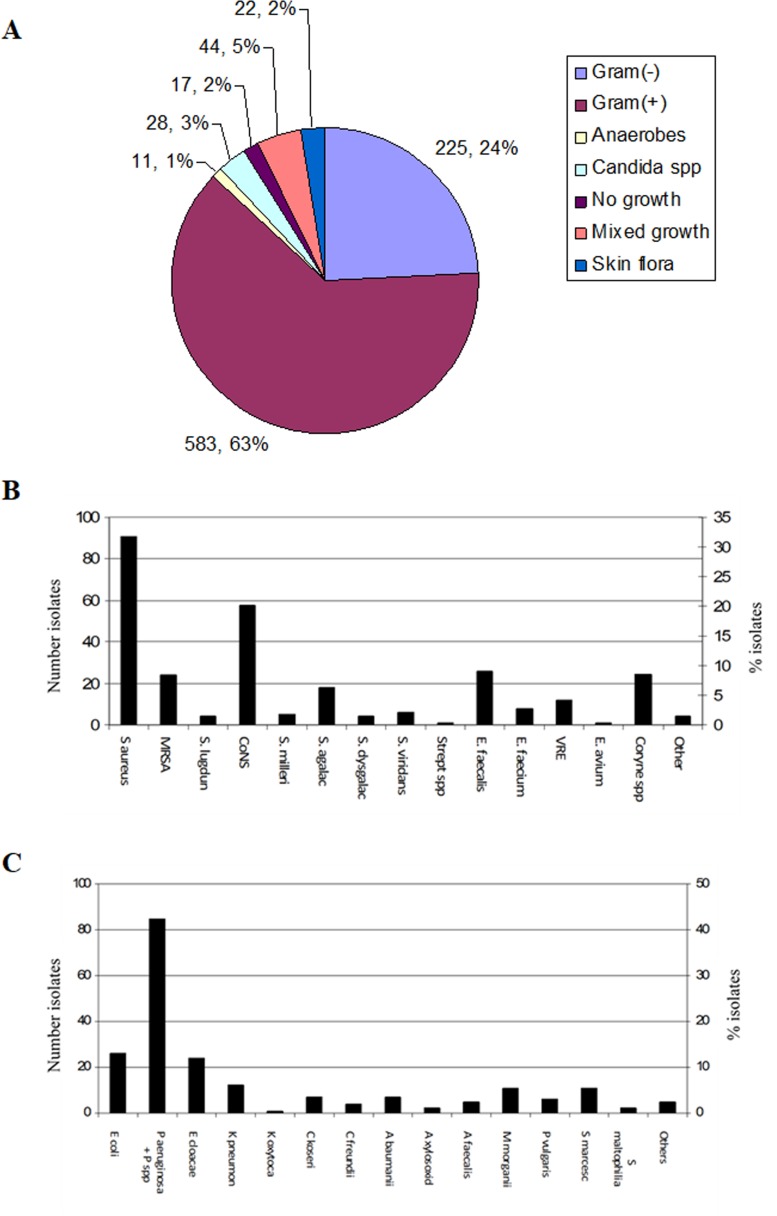
Distribution of microbiological isolates from diabetic foot wounds in patients with osteomyelitis. **A**. Distribution by culture outcome. Anaerobes are not discriminated by Gram stain. **B** and **C**. Distribution of Gram-positive and Gram-negative organisms, respectively.

There were 930 microbiological isolates, mainly represented by Gram-positive organisms (583, 63%; Fig 2A), followed by Gram-negatives (225, 24%). Anaerobes represented only 11 isolates (1%), however the microbiology laboratory does not routinely perform anaerobe culture. No growth, mixed growth and skin flora represented 17 (2%), 44 (5%) and 22 (2%) samples/isolates, respectively. The commonest Gram-positive isolate was *S*.*aureus* (40%), including MRSA, followed by coagulase-negative staphylococcus (CoNS, 20%). Enterococcus, including vancomycin-resistant enterococci (VRE) accounted for 17% of Gram-positive isolates (Fig 2A). *P*.*aeruginosa* was the most frequent Gram-negative isolate (42%), followed by enterobacteriaceae, mainly *E*.*coli* (13%) and *E*.*cloacae* (12%) (Fig 2C).

### Antibiotic resistance

#### Gram-positive organisms

All 67 methicillin-susceptible *S*.*aureus* (MSSA) isolates were sensitive to gentamicin, whereas 10/12 MRSA isolates were gentamicin-sensitive. Nearly 24% of MSSA isolates were sensitive to penicillin but 25% (17/67) were resistant to erythromycin. All *S*.*aureus* isolates, whether MSSA (67) or MRSA (12), were sensitive to linezolid and rifampicin. Ninety-percent of *E*.*faecalis* (9/10) and 100% of *E*.*faecium* (3/3) and VRE (5/5) were resistant to gentamicin. All VRE were also resistant to amoxicillin but all were sensitive to linezolid.

#### Gram-negative organisms

Enterobacteriaceae such as *E*.*coli*, *E*.*cloacae*, *K*.*pneumoniae*, *M*.*morganii*, and *S*.*marcescens* were all meropenem sensitive although one isolate of *M*.*morganii* and one of *E*.*cloacae* were ertapenem resistant. Three out of 55 (5.4%) *P*.*aeruginosa* isolates were meropenem resistant, whereas 7 out of 8 (87.5%) and 2/2 (100%) *A*.*baumanii* isolates were resistant to meropenem and ertapenem, respectively. Resistance to ciprofloxacin was variable ranging from 0% (0/7 isolates) in *M*.*morganii* to 87% (7/8 isolates) in *A*.*baumanii*. *P*.*aeruginosa* resistance to ciprofloxacin was 27% (15/55 isolates). Resistance to gentamicin was generally low, except for *K*.*pneumoniae* (44%, 4/9 isolates) and *A*.*baumanii* (87%, 7/8 isolates).

### Antibiotic therapy

Seventy-six percent (94/124) of the OM cases received co-amoxiclav at some point during treatment, although number of antibiotics used per patient ranged between 1 and 8. Thus, 27% (23) had one antibiotic, whereas 23% (29) had more than 3 antibiotics (Table 3). Appropriate antibiotic switch once antibiotic-susceptibilities were available was done in 87% (108) of patients. Duration of antibiotic therapy was highly variable; about a third of patients (34%) had between 6 weeks and 3 months of antibiotics, 23% had less than 6 weeks, whereas 7% had more than 6 months treatment (Table 4). Of note, 50/117 (43%) patients had been treated with at least one course of antibiotics in the last three months prior to admission.

**Table 3:** Number of antibiotics used per patient.

No of different antibiotics	No of patients (%)
1	**23 (27)**
2	**31 (25)**
3	**22 (18)**
>3	**29 (23)**
**Unknown** -Loss to follow up -Unable to obtain data	**19 (15)**

**Table 4:** Duration of antibiotic therapy.

Antibiotic duration	No of patients (%)
<6 weeks	**35 (23)**
≥6 weeks– 3 months	**42 (34)**
>3 months– 6 months	**15 (12)**
>6 months– 1 year	**6 (5)**
>1 year	**2 (2)**
**Unknown** -Loss to follow up -Treatment for unrelated infection	**24 (19)**

### Outcome

#### Length of stay

**Fig 3:**
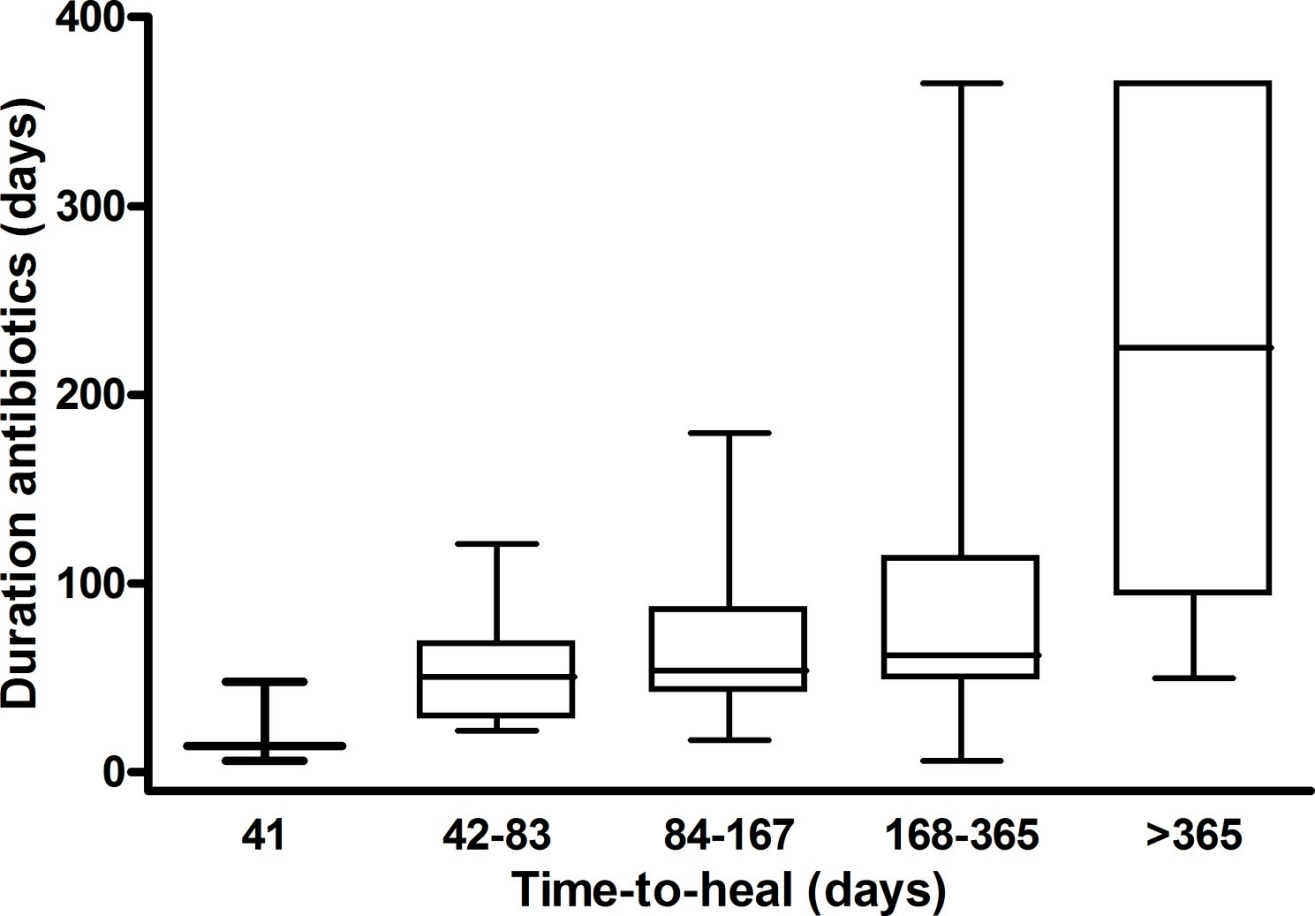
Association between time-to-heal of diabetic foot ulcers and duration of antibiotic treatment. Kruskal-Wallis *p*<0.001.

There were 158 hospital visits, with a visit defined as an admission to hospital with an episode of osteomyelitis from either the same or a different ulcer. Mean number of admissions per case of OM was 1.4. Twenty-three patients were admitted twice and 6 patients were admitted three times. Mean length of hospital stay was 26.1±27.5 days (range 1–225). Most patients (51.3%) had an in-hospital stay of between one week and one month, with nearly 30% staying between 1–3 months (Table 5). Time-to-heal of the DFU was used as a marker of outcome (defined in Methods). This parameter was possible to measure only in 80 patients (64%), due to both loss to follow up and death (45, 36%). Only 3 (2%) patients had a time-to-heal between 1 week and a month, otherwise time-to-heal ranged between one month and more than a year, with the latter represented by 4 (3%) patients. A large number of DFU (28, 22%) took 6–12 months to heal (Table 6), with associated more prolonged antibiotic courses. Those patients with a time-to-heal between 6–12 months had a mean duration of antibiotic use of 85±72 days (range 6–365, median 62). There was association between antibiotic duration and time-to-heal in that the longer the duration of antibiotic therapy the longer the time-to-heal (Fig 3). This association was not possible to analyse in 54 (43%) patients due to either loss to follow up or because unavailability of antibiotic duration data.

**Table 5:** Length of stay in hospital.

Length of stay	No of visits (%)
<1 week	**25 (15.8)**
1 week - ≤1 month	**81 (51.3)**
>1 month - ≤3 months	**47 (29.7)**
>3 months - ≤6 months	**4 (2.5)**
>6 months– 1 year	**1 (0.6)**

**Table 6:** Time-to-heal of diabetic foot ulcers.

Time-to-heal	No of patients (%)
1 week–<1 month	**3 (2)**
1 month—<3 months	**28 (22)**
3 months—<6 months	**17 (14)**
6 months—<1 year	**28 (22)**
≥1 year	**4 (3)**
-Lost to follow up -Patient died -Remained unhealed	**40 (32)** **2 (2)** **3 (2)**

**Fig 4:**
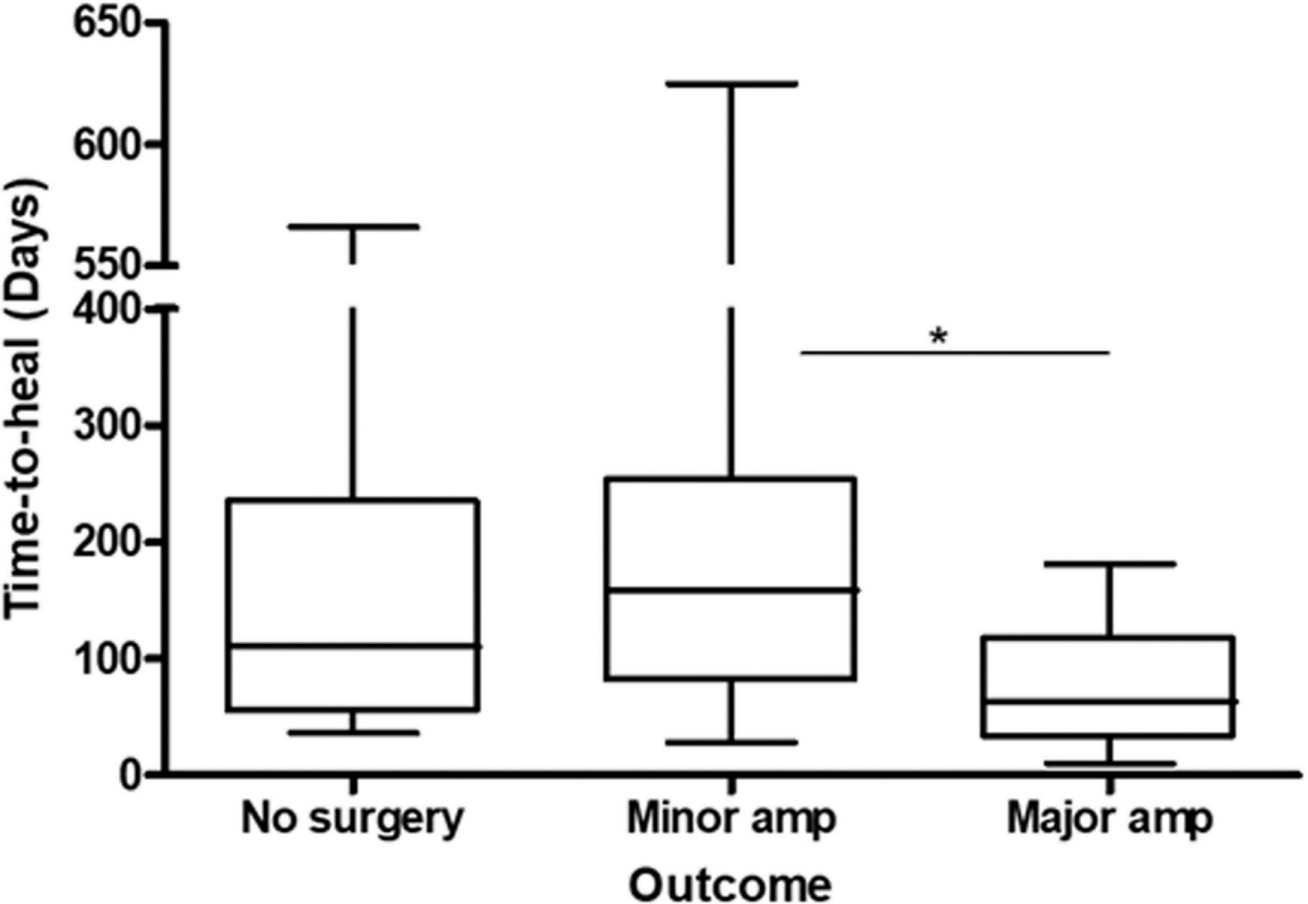
Association between time-to-heal of diabetic foot ulcers and final outcome. *p*<0.02 by Kruskal-Wallis with * = *p*<0.05 by Dunn’s Multiple Comparison test.

Eighteen patients (22%) of 32 who had no surgery had their ulcers healed (4 had a clinical diagnosis of OM, 12 were lost to follow up and two patients died). Fifty-two (65%) patients healed their ulcers only after a minor surgery/partial amputation was performed. Time-to-heal between these two groups was similar: 160 vs 178 days, respectively. Major amputation was needed in 10 (12%) patients with an average time-to-heal of 77 days. Time-to-heal between the three groups was significantly different (Fig 4), which was related to the shorter time-to-heal in those patients with major amputation. The DFU of 3 (4%) patients did not heal during the time frame of this study, and 2 patients died.

Ninety-two percent of the ulcers were in the forefoot and only 10 patients had ulcer in the hindfoot (2 of them also in their forefoot). Hindfoot ulcers usually had poor outcome: 8 of these patients had surgery (2 AKA, 3 BKA, 2 wide debridement), and, upon hospital discharge, 4 were bedbound, 1 was in a wheel chair, and 2 mobilised with one or two prosthesis, depending on whether one or both legs were amputated. Outcome of 3 of these patients was unknown since follow up was carried out at their local hospitals. Only 2 of them had documented evidence of PN, one of whom had BKA secondary to sepsis. Two patients had PVD and were revascularized, but both developed non-healing heel sepsis and ended in BKA, one of them bilateral. Table 7 shows patients’ surgery information, and its outcome.

**Table 7:** Surgery and post-surgery outcome of patients with diabetic foot osteomyelitis.

**Indication for surgery**		n	%
	Dry gangrene/ischaemia	10	8
	Wet gangrene/sepsis	50	40
	Gangrene/necrosis non-specified	9	7
	Non-healing	22	17
	No indication	32	26
**Type of Surgery**^**a**^			
	Debridement	5	4
	Amputation^b^ Partial Transmetatarsal AKA/BKA	61 14 10	49 11 8
	No-surgery	32	26
**Wound location**			
	Forefoot	114	92
	Midfoot	1	1
	Hindfoot	10	8
**Ulcer healing status**			
	Healed	76	61
	Not healed	2	1
	Unknown^c^	38	31
**Discharge destination**			
	Home	104	84
	Rehabilitation centre	8	6
	Other hospital	6	5
	Nursing home	2	2
	Died	2	2
**Mobility post-discharge**			
	Bedbound^d^	6	5
	Wheel-chair	6	5
	Walking with prosthesis^e^	3	2
	Walking without prosthesis	67	54
	Unknown^c^	38	31

^a^: Type of surgery specified is the latest performed in a patient

^b^: Partial amputation refers to single or multiple toe or metatarsal amputation/excision.

^c^: Lost to follow up due to further management at patient’s local hospital

^d^: Four of the bedbound patients had hindfoot ulcer and had undergone AKA or BKA

^e^: Prosthesis was either for one leg or for both legs. Two of these patients had hindfoot ulcer

Presence of PVD and PN and the effect of revascularisation on wound outcome was analysed. Seventy-five (65%) and 40 (34%) patients had PVD and PN, respectively. Of these, 23 (20%) had both complications. Revascularisation was performed in 50 of the 75 patients (67%) with PVD. Forty (80%) of these revascularised patients underwent amputation (28 partial amputation, 5 transmetatarsal, 7 BKA). Thirteen out of 16 patients (81%) with PVD who did not undergo revascularisation, had surgery (8 partial amputation, 4 transmetatarsal, 1 debridement). Nine out of 23 (39%) patients who had both PVD and PN underwent surgery, regardless of whether they had revascularisation or not (6 partial amputation, 2 transmetatarsal, 1 BKA).

## Discussion

We retrospectively analysed the management of 124 DFOM cases by an MDT during 2015 at a tertiary referral Hospital in London, UK.

Osteomyelitis was diagnosed in 42% of 275 patients with DFU seen during 2015. Diagnosis used a combination of clinical findings, including probe-to-bone test, and radiological and microbiological information. Since plain X-ray was used in 100 cases (80.6%), and only 54% of these showed evidence of OM, it appears that diagnosis heavily relied on clinical impression. This was despite the probe-to-bone-test, an adjunct to clinical diagnosis, been performed just in 25% of patients. This may, however, represent an underestimate since this test was not always documented. It is clear from the data that MRI played little part in diagnosing OM, since this was performed only in 28% of cases. A large proportion of patients (~40%) had a bone biopsy, which contributed to confirm the diagnosis.

Inflammatory markers, such as WCC and CRP, are useful to determine the likely presence of sepsis, either locally or systemically. Our data compared the CRP and WCC values of those patients who did not have surgery with those who did. CRP was significantly higher in those patients who had surgery (Fig 1A), specifically in those whose sepsis was the indication for surgery (Fig 1C). Some of these patients may have arrived in the hospital with signs of systemic sepsis, hence surgery was an imminent decision. However, for those with ischaemia who may also have had urgent surgery, the CRP was no raised. Fleischer et al, showed that CRP greater than 3.2 mg/dL and an ulcer depth greater than 3mm were highly sensitive in the diagnosis of DFO [8]. However, the CRP role to predict indication for surgery was not included in that study. WCC was also significantly raised in patients who had surgery due to sepsis (Fig 1D). It is expected that the clinical setting helps to decide on whether the patient needs surgery at some point [3], and our data suggest that CRP and WCC may be good indicators of this need. It is however important to highlight that up to 50% of patients with deep foot infection will not have raised leukocyte counts [9], which means that normal results do not rule out infection.

A significant proportion of patients (25%) was transferred from other hospitals. This is relevant, because once patients improve clinically they are returned to the referral hospital where DFOM management may vary considerably. This may reduce the availability of follow up information and impact on some of the overall outcome data. These drawbacks may be less of an issue in hospitals with captive populations where there are fewer cross-boundary referrals as suggested by Krishnan, et al [10].

The most common location of OM was the forefoot (92%), which is consistent with previous studies [11]. An observational study that prospectively assessed 291 patients with DF infection admitted to 38 hospital centres found that nearly 50% of patients had OM with most infections involving the toes (45%) or forefoot (34%) [12]. These findings are not surprising since most ulcers develop on the plantar surface, usually under the metatarsal heads or on the toes; plantar ulcers occur most frequently at sites of high plantar pressure [13]. A great proportion of our patients had a pre-existing ulcer (47%) at the time of hospitalisation, which is similar to that of 40% described by others [12]. Although in small numbers (ten), hindfoot location of the wound had a poor outcome. Most of these patients had surgery and underwent either above or below knee amputation with subsequent poor mobility status (bedbound or wheelchair users). Only one of these patients was recorded as having PN and two had PVD with revascularisation.

Sixty percent of patients had PVD, which is higher than the nearly 50% reported by Prompers et al [14], 32% had PN, with 18% having both complications. Revascularisation was performed in 67% of those patients with PVD. Of note, 80% of the patients who underwent revascularisation had surgery, of which 7 had BKA. This percentage was similar to that of 81% operation rate in those who were not revascularised. Although revascularisation may be important for a critically ischaemic limb, it may not necessarily have a direct beneficial effect on the infection process [15].

Microbiological diagnosis of OM was performed using swab, tissue and bone sampling, or a combination of these. Swabs predominated (43.2% of 414 samples in total) but we acknowledge the questionable utility of this type of sample [3, 16, 17]. Direct comparisons of swab sample performance with that of bone and tissue culture were not possible because of differing standard processing and reporting for these microbiology samples (our laboratory does not work up routine swab samples that have >3 organisms).

Bone biopsy represented nearly 40% of samples, most of them taken in theatre (86% out of 66 OM cases for which it was possible to establish the place of sampling). Clearly, the MDT put emphasis on obtaining bone biopsy (see Table 2), which is the gold standard for OM diagnosis [5, 9].

As expected, most isolates were Gram-positive organisms (63%). *S*.*aureus* and CoNS accounted for around 50% of these isolates. Only 7% of Gram-positive isolates were MRSA, confirming the trend of low MRSA incidence in England [18]. Several other Gram-positive cocci were also isolated, all in small numbers. These isolates have different levels of pathogenicity and may represent colonising flora, although *S*.*agalactiae* is by itself highly pathogenic.

*P*.*aeruginosa* was the commonest Gram-negative isolate (>40%). This was unexpected, because, although commonly isolated from DFU, *P*.*aeruginosa* is not usually the most frequently isolated organism at least in temperate countries [19–22]. It has been suggested that *P*.*aeruginosa* is more frequently isolated from DFU in warm climates [3]. However, findings of a study in a tropical country were not different to those found in temperate countries [23]. We do not have an explanation for the high numbers of *P*.*aeruginosa* in DFU in our patients, but whether this has an impact on treatment and prognosis deserves further analysis. *A*.*baumanii* was isolated from a small number of patients; this is a troublesome organism, since it is usually highly resistant to antibiotics.

Sensitivity of *S*.*aureus* to antibiotics was high, although 25% of isolates were resistant to macrolides. This is important because clindamycin, which shares resistance mechanisms with macrolides, may be used empirically to treat infected DFU. *S*.*aureus* resistance to clindamycin was 16.7%. Both MRSA and VRE were sensitive to linezolid. This antibiotic may become the sole treatment option, particularly when VRE is the cause of infection. There are emergent VRE isolates that are also resistant to linezolid and daptomycin [24] and it is expected that linezolid resistant VRE may appear in the future. The limitation of using linezolid in diabetes patients is their higher propensity to the neuropathic side effects of this antibiotic [25], although this side effect was not reported by Lipsky et al [26].

Antibiotic usage is fundamental in DFOM therapy. Multiple antibiotics are commonly used, and duration tends to be long, either continuously or intermittently. Good antibiotic stewardship is important, emphasising the role of microbiologists in the MDT. In our study, patients received between 1–8 different antibiotics, with nearly 25% receiving >3 antibiotics during their therapy (Table 3). A high proportion of patients (75%) received co-amoxiclav at some point. This reflects either the simplicity of selecting a broad-spectrum antibiotic or that it represents a good antibiotic option for stepdown therapy. Of note, antibiotic switch was done in 87% of patients once microbiology results were available. This is much higher than the 56% reported by Richard, JL, et al [12].

Duration of antibiotic therapy was long; only 23% of patients had ≤6 weeks therapy. Fifty-seven percent of patients had antibiotic treatment lasting between 6 weeks and 6 months and 43% were treated with at least one course of antibiotics in the last three months prior to admission, the latter matching figures published by Richard JL, et al [12]. Interestingly, only 5 patients developed *C*.*difficile* infection despite long-term antibiotic treatment. It is noteworthy that this infection is unusual in diabetic patients [27], suggesting some kind of unknown mechanisms of tolerance to infection, which deserves further investigation. Of note, metformin was associated with a significant reduction in *C*.*difficile* infection in diabetic patients [28].

The outcome of DFOM is measurable through parameters such as time of hospitalisation, time-to-heal of ulcer, and whether patients undergo amputation [3]. Mean length of hospital stay was 26.1 days, similar to that of 3 weeks published elsewhere [12]. More than 67% of patients stayed in hospital for up to a month and nearly 30% stayed between 1–3 months, both accounting for about 97% of the patients. Whether the length of hospital stay was related to other comorbidities, severity of the DFU, or need for lengthy IV antibiotic therapy was not scrutinised in this work but it may be worth investigating further. The lengthy hospital stay of these patients, represents a socio-economic burden to the health system, as well as to the patient and patient’s family, so devising mechanisms to shorten this hospitalisation time will certainly contribute to lessen this burden [29].

Only 2% of patients’ wounds healed within a month, whereas 36% took between 3–12 months to heal (Table 6). The Eurodiale study group found 23% of wounds unhealed after 1 year of follow up [30]. We found a statistically significant association between time-to-heal and duration of antibiotics (Fig 3), so that the longer the time-to-heal the longer the antibiotic therapy. The reasons for this are not clear from the information we gathered, however peripheral artery disease and infection that ensue in these patients are among independent predictors of non-healing [30].

Surgery is an important part in the management of DF ulcers. This encompasses from simple ulcer debridement through revascularisation procedures to amputation. The latter may be either minor or major. One of the strengths of the DF MDT analysed here, is the role of the vascular surgeons. These are closely involved in case discussions and decisions regarding the need for revascularisation of severely diseased lower limb blood vessels, or instead the use of radical measures such as minor or major amputation. In our study, 72% of the patients had minor (60%) and major (12%) lower limb amputations, which is higher than the accumulated 54% observed by Richard et al [12]. We show that only major amputation significantly lowered the wound’s time-to-heal (Fig 4). Minor amputations did not affect this time, which may be explained by persistence of infected bone, or architectural reorganisation of the foot altering the foot biomechanics generating a vicious circle of high pressure sites associated with skin breakdown and re-ulceration [3]. These results suggest that conservative management of DFOM is perhaps, at least in some cases, only prolonging the inevitable, which is major amputation.

A clear example of the invaluable benefit of the DF MDT is the sharp decrease in amputation rate during an 11-year period in a district hospital in the UK thanks to improvements in foot care services delivered by a DF MDT [10].

There is no doubt that more work is needed that helps to understand the complexity of DFOM. The best way to start this process would be by developing well-coordinated DF MDTs with regular auditing of their performance, so lessons can be learned, and improvements can be implemented.

## References

[1] World Health Organisation. Global Report on Diabetes. Geneva, Switzerland: WHO, 2016.

[2] National Institue for Health and Care Excellence (NICE). Diabetic Foot Problems. Prevention and Management. 2016. 32045177

[3] Lipsky BA, Berendt AR, Cornia PB, Pile JC, Peters EJ, Armstrong DG, et al 2012 Infectious Diseases Society of America clinical practice guideline for the diagnosis and treatment of diabetic foot infections. Clin Infect Dis. 2012;54(12):e132–73. Epub 2012/05/24. 10.1093/cid/cis346 . 22619242

[4] Lipsky BA. Medical treatment of diabetic foot infections. Clin Infect Dis. 2004;39 Suppl 2:S104–14. Epub 2004/08/13. 10.1086/383271 . 15306988

[5] Jeffcoate WJ, Lipsky BA. Controversies in diagnosing and managing osteomyelitis of the foot in diabetes. Clin Infect Dis. 2004;39 Suppl 2:S115–22. Epub 2004/08/13. 10.1086/383272 . 15306989

[6] Royal College of Physicians. National Early Warning Score (NEWS): Standardising the assessment of acute illness severity in the NHS. Report of a working party. London: RCP, 2012.

[7] Royal College of Physicians. National Early Warning Score (NEWS) 2: Standardising the assessment of acute-illness severity in the NHS. Upated report of a working party. London: RCP, 2017.

[8] Fleischer AE, Didyk AA, Woods JB, Burns SE, Wrobel JS, Armstrong DG. Combined clinical and laboratory testing improves diagnostic accuracy for osteomyelitis in the diabetic foot. J Foot Ankle Surg. 2009;48(1):39–46. Epub 2008/12/27. 10.1053/j.jfas.2008.09.003 . 19110158

[9] Williams DT, Hilton JR, Harding KG. Diagnosing foot infection in diabetes. Clin Infect Dis. 2004;39 Suppl 2:S83–6. Epub 2004/08/13. 10.1086/383267 . 15306984

[10] Krishnan S, Nash F, Baker N, Fowler D, Rayman G. Reduction in diabetic amputations over 11 years in a defined U.K. population: benefits of multidisciplinary team work and continuous prospective audit. Diabetes Care. 2008;31(1):99–101. Epub 2007/10/16. 10.2337/dc07-1178 . 17934144

[11] Lipsky BA, Aragon-Sanchez J, Diggle M, Embil J, Kono S, Lavery L, et al IWGDF guidance on the diagnosis and management of foot infections in persons with diabetes. Diabetes Metab Res Rev. 2016;32 Suppl 1:45–74. Epub 2015/09/20. 10.1002/dmrr.2699 . 26386266

[12] Richard JL, Lavigne JP, Got I, Hartemann A, Malgrange D, Tsirtsikolou D, et al Management of patients hospitalized for diabetic foot infection: results of the French OPIDIA study. Diabetes Metab. 2011;37(3):208–15. Epub 2010/12/21. 10.1016/j.diabet.2010.10.003 . 21169044

[13] Ulbrecht JS, Cavanagh PR, Caputo GM. Foot problems in diabetes: an overview. Clin Infect Dis. 2004;39 Suppl 2:S73–82. Epub 2004/08/13. 10.1086/383266 . 15306983

[14] Prompers L, Huijberts M, Apelqvist J, Jude E, Piaggesi A, Bakker K, et al High prevalence of ischaemia, infection and serious comorbidity in patients with diabetic foot disease in Europe. Baseline results from the Eurodiale study. Diabetologia. 2007;50(1):18–25. Epub 2006/11/10. 10.1007/s00125-006-0491-1 . 17093942

[15] Uckay I, Aragon-Sanchez J, Lew D, Lipsky BA. Diabetic foot infections: what have we learned in the last 30 years? Int J Infect Dis. 2015;40:81–91. Epub 2015/10/16. 10.1016/j.ijid.2015.09.023 . 26460089

[16] Slater RA, Lazarovitch T, Boldur I, Ramot Y, Buchs A, Weiss M, et al Swab cultures accurately identify bacterial pathogens in diabetic foot wounds not involving bone. Diabet Med. 2004;21(7):705–9. Epub 2004/06/24. 10.1111/j.1464-5491.2004.01221.x . 15209762

[17] Nelson EA, Wright-Hughes A, Brown S, Lipsky BA, Backhouse M, Bhogal M, et al Concordance in diabetic foot ulceration: a cross-sectional study of agreement between wound swabbing and tissue sampling in infected ulcers. Health Technol Assess. 2016;20(82):1–176. Epub 2016/11/09. 10.3310/hta20820 27827300 PMC5116580

[18] Public Health England. Annual Epidemiological Commentary: Mandatory MRSA, MSSA and E. coli bacteraemia and C. difficile infection data 2016/17. 2017.

[19] Wheat LJ, Allen SD, Henry M, Kernek CB, Siders JA, Kuebler T, et al Diabetic foot infections. Bacteriologic analysis. Arch Intern Med. 1986;146(10):1935–40. Epub 1986/10/01. . 3767539

[20] Sapico FL, Canawati HN, Witte JL, Montgomerie JZ, Wagner FW Jr., Bessman AN. Quantitative aerobic and anaerobic bacteriology of infected diabetic feet. J Clin Microbiol. 1980;12(3):413–20. Epub 1980/09/01. 7217335 10.1128/jcm.12.3.413-420.1980 PMC273599

[21] Louie TJ, Bartlett JG, Tally FP, Gorbach SL. Aerobic and anaerobic bacteria in diabetic foot ulcers. Ann Intern Med. 1976;85(4):461–3. Epub 1976/10/01. . 970773 10.7326/0003-4819-85-4-461

[22] Giurato L, Meloni M, Izzo V, Uccioli L. Osteomyelitis in diabetic foot: A comprehensive overview. World J Diabetes. 2017;8(4):135–42. Epub 2017/05/04. 10.4239/wjd.v8.i4.135 28465790 PMC5394733

[23] Zuluaga AF, Galvis W, Jaimes F, Vesga O. Lack of microbiological concordance between bone and non-bone specimens in chronic osteomyelitis: an observational study. BMC Infect Dis. 2002;2:8 Epub 2002/05/23. 10.1186/1471-2334-2-8 12015818 PMC115844

[24] Chacko KI, Sullivan MJ, Beckford C, Altman DR, Ciferri B, Pak TR, et al Genetic Basis of Emerging Vancomycin, Linezolid, and Daptomycin Heteroresistance in a Case of Persistent Enterococcus faecium Bacteremia. Antimicrob Agents Chemother. 2018;62(4). Epub 2018/01/18. 10.1128/AAC.02007-17 29339387 PMC5913925

[25] Swaminathan A, du Cros P, Seddon JA, Mirgayosieva S, Asladdin R, Dusmatova Z. Peripheral neuropathy in a diabetic child treated with linezolid for multidrug-resistant tuberculosis: a case report and review of the literature. BMC Infect Dis. 2017;17(1):417 Epub 2017/06/14. 10.1186/s12879-017-2499-1 28606115 PMC5469058

[26] Lipsky BA, Itani KM, Weigelt JA, Joseph W, Paap CM, Reisman A, et al The role of diabetes mellitus in the treatment of skin and skin structure infections caused by methicillin-resistant Staphylococcus aureus: results from three randomized controlled trials. Int J Infect Dis. 2011;15(2):e140–6. Epub 2010/12/08. 10.1016/j.ijid.2010.10.003 . 21134775

[27] Collier A, McLaren J, Godwin J, Bal A. Is Clostridium difficile associated with the '4C' antibiotics? A retrospective observational study in diabetic foot ulcer patients. Int J Clin Pract. 2014;68(5):628–32. Epub 2014/02/07. 10.1111/ijcp.12347 24499256 PMC4238420

[28] Eliakim-Raz N, Fishman G, Yahav D, Goldberg E, Stein GY, Zvi HB, et al Predicting Clostridium difficile infection in diabetic patients and the effect of metformin therapy: a retrospective, case-control study. Eur J Clin Microbiol Infect Dis. 2015;34(6):1201–5. Epub 2015/02/18. 10.1007/s10096-015-2348-3 . 25686730

[29] Prompers L, Huijberts M, Schaper N, Apelqvist J, Bakker K, Edmonds M, et al Resource utilisation and costs associated with the treatment of diabetic foot ulcers. Prospective data from the Eurodiale Study. Diabetologia. 2008;51(10):1826–34. Epub 2008/07/24. 10.1007/s00125-008-1089-6 . 18648766

[30] Prompers L, Schaper N, Apelqvist J, Edmonds M, Jude E, Mauricio D, et al Prediction of outcome in individuals with diabetic foot ulcers: focus on the differences between individuals with and without peripheral arterial disease. The EURODIALE Study. Diabetologia. 2008;51(5):747–55. Epub 2008/02/26. 10.1007/s00125-008-0940-0 18297261 PMC2292424

